# Recent advances in the understanding and management of *Klebsiella pneumoniae*

**DOI:** 10.12688/f1000research.11532.1

**Published:** 2017-09-27

**Authors:** David P. Calfee

**Affiliations:** 1 Weill Cornell Medicine, New York, USA

**Keywords:** Klebsiella pneumoniae, Enterobacteriaceae, K1 capsular serotype

## Abstract

*Klebsiella pneumoniae*, a gram-negative bacillus of the Enterobacteriaceae family, is a component of the normal human microbiota and a common cause of community- and healthcare-associated infections. The increasing prevalence of antimicrobial resistance among *K. pneumoniae* isolates, particularly among those causing healthcare-associated infections, is an important public health concern. Infections caused by these multidrug-resistant organisms, for which safe and effective antimicrobial therapy options are extremely limited, are associated with poor outcomes for patients. The optimal approach to the treatment of infections caused by these multidrug-resistant strains remains undefined, and treatment decisions for an individual patient should be based on a number of organism- (for example, minimum inhibitory concentration) and patient-specific (for example, site of infection) factors. The emergence of pandrug-resistant strains of *K. pneumoniae* highlights the critical need for consistent implementation of effective strategies for prevention of transmission and infection and for the development of new antimicrobials with activity against these emerging pathogens.

## Epidemiology

*Klebsiella pneumoniae*, a member of the Enterobacteriaceae family of gram-negative bacilli, is part of the normal microbiome of many humans. Among 242 healthy volunteer participants in the National Institutes of Health Human Microbiome Project, *K. pneumoniae* was identified in 3.8% and 9.5% of stool and nasal samples, respectively ^1^. Colonization was also detected at cutaneous, oral, and vaginal sites. There appears to be substantial geographic variation in the proportion of persons in whom the normal intestinal microbiota includes *K. pneumoniae*. For example, in a Korean study, *K. pneumoniae* was detected in stool samples obtained from 21.1% of 1,174 healthy persons at least 16 years of age ^2^.

### Community-acquired *K. pneumoniae* infections

The most commonly identified community-associated infections caused by *K. pneumoniae* include urinary tract infections (UTIs), pneumonia, and liver abscesses. Community-acquired liver abscesses caused by hypervirulent strains (for example, the K1 capsular serotype) of *K. pneumoniae* have become increasingly common among persons without underlying hepatobiliary disease, particularly in Asia’s Pacific Rim. One potential explanation for these geographic differences in the incidence of disease is a higher rate of intestinal carriage of *K. pneumoniae* of the K1 serotype among persons living in this region. In the previously mentioned Korean study, among the 21.1% of healthy adults who had fecal carriage of *K. pneumoniae*, 23% were carriers of serotype K1 ^2^. Interestingly, the rates of carriage of the K1 serotype were 5.6% among adults older than 25 years of age and 0% among those 16–25 years of age ( *P*=0.007), and the prevalence of the K1 serotype was higher among isolates from ethnic Koreans residing in Korea than among those from persons residing in other countries (24.1% versus 5.6%). These findings suggest that environmental factors, such as diet, may play a role in the epidemiologic differences observed with regard to this pathogen ^2^.

In the laboratory, these hypervirulent strains are phenotypically characterized by hypermucoviscosity often defined by a positive “string test” in which a viscous string of more than 5 mm in length forms when bacterial colonies on an agar plate are stretched by an inoculation loop. Acquisition of an *rmpA* gene-containing plasmid has been associated with this phenotype through *rmpA*-mediated increases in capsule production. Explanations for the increased virulence of these strains include resistance to complement and neutrophil-mediated bactericidal activity ^3^. Liver abscesses caused by these particular serotypes are associated with an increased risk of hematogenous dissemination to other sites (for example, lung, eye, and central nervous system) and with relatively high rates of morbidity and mortality (4–8%). Evaluation for hepatic abscess should be considered in persons presenting with *K. pneumoniae* bloodstream infection, endophthalmitis, or central nervous system infection without another explanation, particularly among persons with epidemiologic risk factors (for example, residence in or travel to endemic regions of Asia). Extended-spectrum beta-lactamases (ESBLs) and AmpC beta-lactamases are fortunately not common among these strains but have been reported ^4, 5^.

### Healthcare-associated *K. pneumoniae* infections

Although *K. pneumoniae* is a relatively infrequent cause of community-acquired infections, it is a common cause of healthcare-associated infections. In fact, *Klebsiella* species (that is, *K. pneumoniae* and *K. oxytoca*) were the third most common pathogens reported among all cases of central line-associated bloodstream infection (CLABSI), catheter-associated urinary tract infection (CAUTI), ventilator-associated pneumonia, and surgical site infection reported to the National Healthcare Safety Network (NHSN) of the Centers for Disease Control and Prevention (CDC) between 2011 and 2014, representing 7.7% of all reported pathogens ^6^. Over the past few years, several outbreaks of infection caused by multidrug-resistant strains of *K. pneumoniae* and other Enterobacteriaceae associated with the use of contaminated duodenoscopes have been reported. These outbreaks have been attributed at least in part to the complex design of duodenoscopes that renders them more difficult to adequately clean and disinfect as compared with endoscopes with simpler designs ^7– 10^. Although these duodenoscope-related infections have largely been associated with carbapenem-resistant *K. pneumoniae* (CRKP) and other carbapenem-resistant Enterobacteriaceae (CRE), these infections may represent just the tip of the iceberg of duodenoscope-related infections as infections caused by organisms with less notable resistance patterns may be less likely to trigger further investigation of a possible device-associated infection.

The increased incidence of *K. pneumoniae* infection among persons with current or recent healthcare exposures appears to be due at least in part to increased rates and/or bacterial burdens of gastrointestinal carriage of the organism, which has been identified as a risk factor for subsequent *K. pneumoniae* infection. For example, in a prospective cohort study of 498 patients, rates of carriage of *K. pneumoniae*, as detected by rectal and throat samples collected shortly after admission to the intensive care unit (ICU), were approximately 6% among patients admitted directly from the community and 19% among patients with recent healthcare contacts ^11^. In that study, *K. pneumoniae* colonization was found to be a significant risk factor for subsequent *K. pneumoniae* infection while in the ICU, and 16% of carriers versus 3% of non-carriers developed infection and approximately half of *K. pneumoniae* infections came from the patients’ own microbiota. Similarly, another study of patients hospitalized in ICUs and oncology wards found a prevalence of rectal carriage of *K. pneumoniae* of 23% ^12^. Among those identified as carriers, 5.2% subsequently developed *K. pneumoniae* infection (versus 1.3% of non-colonized patients, odds ratio (OR) 4.06, *P*<0.001). Finally, 27% of ICU patients identified as asymptomatic carriers of CRKP through perianal swab specimens collected as part of an active surveillance program subsequently developed CRKP bloodstream infection during their index hospitalization ^13^.

## Antimicrobial resistance

Antimicrobial resistance among clinical isolates of *K. pneumoniae* has become an increasingly serious problem over the past 20 years, and there are multiple examples of rapid global dissemination of novel mechanisms of resistance, such as the *K. pneumoniae* carbapenemase (KPC) and the New Delhi metallo-beta-lactamase (NDM). The problem of antimicrobial resistance is particularly prevalent among healthcare-associated *K. pneumoniae* isolates. Among *K. pneumoniae* and *K. oxytoca* isolates from CLABSI and CAUTI cases reported to the NHSN in 2014, rates of resistance ranged from 22.5 to 24.1% for extended-spectrum cephalosporins and 9.5 to 10.9% for carbapenems ^6^. Among these same isolates, rates of multidrug resistance, defined as resistance to at least one drug in at least three of five classes, ranged from 14.6 to 17.2%.

The growing problem of antimicrobial resistance among gram-negative organisms such as *K. pneumoniae* has led some to warn of the potential for a return to the pre-antibiotic era and the arrival of a “post-antibiotic era” in which we no longer have antibiotics effective for the treatment of infections caused by relatively common bacterial pathogens ^14^. As one example, a recent report described a patient in Nevada who died in September 2016 from septic shock due to pandrug-resistant *K. pneumoniae* infection ^15^. The isolate was non-susceptible to all 27 available antimicrobial drugs. This particular case occurred in a region with a relatively low prevalence of CRE. The isolate was subsequently found to carry the NDM gene, suggesting that the organism may have been acquired during the patient’s previous healthcare exposures in India and highlighting the importance of international travel, globalization, and healthcare exposures in the epidemiology of antimicrobial resistance.

### Carbapenem resistance

In a 2013 report, the CDC identified CRE, the most common of which is *K. pneumoniae*, as one of the top three urgent antimicrobial resistance threats ^16^. This report estimated that, in the US each year, there are 7,900 infections and 520 deaths due to carbapenem-resistant *Klebsiella* species. KPC is the most common mechanism of carbapenem resistance among *K. pneumoniae* isolates in the US. As of January 2017, KPC-producing CRE had been reported to the CDC from 48 states in the US ^17^. KPC-producing CRE typically also have genes that confer resistance to several other classes of antimicrobials, resulting in these organisms displaying a multidrug-resistant phenotype. Among CRE isolates reported to the CDC as of January 6, 2017, carbapenemases other than KPC that have been identified include NDM (n=175), oxacillinase-48-type carbapenemase (OXA-48, n=73), Verona integron-encoded metallo-beta-lactamase (VIM, n=27), and imipenemase metallo-beta-lactamase (IMP, n=11). There are other mechanisms of carbapenem resistance, including hyperproduction of the AmpC beta-lactamase with coexisting porin mutations. In some regions outside the US, non-KPC mechanisms of resistance predominate.

### Polymyxin resistance

The polymyxins—polymyxin E (or colistin) and polymyxin B—serve as drugs of last resort for treatment of infections caused by CRKP and other multidrug-resistant gram-negative pathogens; however, resistance to the polymyxins is a well-described phenomenon and has become increasingly common in some areas, and a rate of 27% was reported among KPC-producing *K. pneumoniae* isolates from five Italian hospitals in 2013 ^18^. Between 2009 and 2012, colistin resistance was detected in 2.7% of *Klebsiella* isolates obtained from patients hospitalized with pneumonia at one of 28 medical centers in the US ^19^. Polymyxin resistance has been reported as an independent predictor of 14-day mortality among patients with KPC-producing *K. pneumoniae* infections ^18^.

Resistance to polymyxin is most commonly chromosomally mediated and this is typically due to post-translational modification of bacterial outer-membrane lipopolysaccharide through the addition of cationic groups as a result of mutations in the *mgrB* regulatory gene ^20– 24^. Previous treatment with colistin has been associated with infection due to colistin-resistant KPC-producing *K. pneumoniae* ^25^; however, the majority of patients with colistin-resistant KPC- *K. pneumoniae* bloodstream infection in one study had not previously received colistin, indicating that other factors, such as healthcare-associated transmission of resistant strains, likely also play a role. More recently, a plasmid-mediated gene ( *mcr-1*) that confers resistance to polymyxin was discovered. The gene has been identified among Enterobacteriaceae (most commonly *E. coli* but also other genera, including *Klebsiella*) isolated from animal and human sources. The initial report from China in November 2015 includes isolates obtained as early as 2011 ^26^. In that investigation, the rates of prevalence of the *mcr-1* gene were 20.6% among *E. coli* isolates obtained from pigs at the time of slaughter, 14.9% among *E. coli* isolates obtained from retail chicken and pork, and 1.4% and 0.7% among *E. coli* and *K. pneumoniae* isolates, respectively, from human inpatients. Since that report, *mcr-1* has been detected in food animals, food, or humans (or a combination of these) in other Asian countries, Europe, Africa, North America, and South America ^27, 28^. As of March 30, 2017, 14 *mcr-1*-positive isolates, including 12 human and two animal isolates, had been reported from 11 US states ^29^. Investigators have demonstrated successful *in vitro* transfer of the *mcr-1* gene between *E. coli* isolates by conjugation and from *E. coli* to *E. coli*, *K. pneumoniae*, and *Pseudomonas aeruginosa* by transformation ^26^, raising significant concerns about the possibility of acquisition of this mobile genetic element by organisms that already possess other drug-resistance determinants (for example, KPC) resulting in pandrug-resistant bacteria.

Accurate assessment of polymyxin susceptibility among multidrug-resistant strains of *K. pneumoniae* is important for both clinical and epidemiologic purposes. Polymyxin susceptibility testing is challenging for several reasons (for example, poor diffusion of polymyxins into agar and adherence of polymyxins to components of some materials used in the laboratory) and these challenges can lead to unacceptably high error rates when methods such as disk and agar gradient diffusion are used. The Clinical Laboratory Standards Institute/European Committee on Antimicrobial Susceptibility Testing (CLSI/EUCAST) Joint Polymyxin Breakpoints Working Group recently identified broth microdilution as the reference method for testing colistin susceptibility ^30^. No specific statements or recommendations for polymyxin B susceptibility testing were made, but many of the same limitations and challenges as noted for colistin are applicable to polymyxin B as well.

## Outcomes

Infections caused by multidrug-resistant strains of *K. pneumoniae* have been associated with substantial morbidity, mortality, and cost. The attributable length of hospital stay due to CRE infections has been reported to range from 4 to 21 days, depending on the site of infection ^31^. Reported rates of attributable mortality have ranged from 10 to 70%, again varying substantially among reports and among different sites of infection. A multinational group of investigators recently developed and validated a predictive model of 14-day mortality in patients with monomicrobial bloodstream infection caused by carbapenemase-producing Enterobacteriaceae ^32^. The final model, which exhibited an area under the receiver operating characteristic curve of 0.8 (95% confidence interval (CI) 0.73–0.88), included five factors that could be assessed when antimicrobial susceptibility testing results become available: severe sepsis or shock at presentation, Pitt bacteremia score of at least 6, Charlson comorbidity index of at least 2, source of bloodstream infection other than urinary or biliary tract, and inappropriate empiric and early targeted antimicrobial therapy. The cost of a single CRE infection has been estimated to range from $22,484 to $66,031 for hospitals and from $37,778 to $83,512 for society ^31^. Given 9,418 CRE infections in the US per year, the annual costs could be as high as $334 million and $1.59 billion for hospitals and society, respectively, with a loss of up to 12,420 quality-adjusted life years.

## Treatment of infections caused by carbapenem-resistant *K. pneumoniae*

The optimal antimicrobial treatment of infections caused by CRKP remains to be determined and for any individual patient is likely dependent upon a number of patient-specific (for example, site of infection) and organism-specific (for example, minimum inhibitory concentration [MIC] for individual antimicrobial agents) factors. As noted above, mortality rates for these infections remain unacceptably high with currently available therapy. In addition to antimicrobial agents, other therapeutic interventions such as source control should be used whenever possible in order to maximize the likelihood of a successful outcome. Strategies for, and the challenges and limitations associated with, the use of the currently available antimicrobial agents are thoroughly reviewed in previously published articles ^33^, but a few topics are worthy of brief discussion here.

### Combination therapy

Some but not all retrospective studies have found an association between combination therapy and lower mortality as compared with treatment with single-drug treatment regimens. One of the more common combination therapy regimens is that of a polymyxin plus an optimized dosing regimen of a carbapenem (for example, meropenem or doripenem). Some observational studies have reported better outcomes among patients who received polymyxin-carbapenem combination therapy, particularly in patients or settings (or both) in which *K. pneumoniae* isolates demonstrated relatively low-level carbapenem resistance (MIC ≤8–16) ^18, 34^. Retrospective studies that have assessed this strategy among patients infected with CRKP with high-level carbapenem resistance have generally not demonstrated additional benefit ^35, 36^. These MIC-dependent differences in clinical outcomes are supported by *in vitro* testing that has demonstrated synergy between bactericidal activity of polymyxin B plus meropenem against KPC-2-producing *K. pneumoniae* isolates with meropenem MICs of not more than 16 μg/mL ^37^. Similarly, exposure to this combination of agents produced morphological changes in bacterial isolates that decreased as the meropenem MIC increased. The pharmacodynamic effects of combination therapy decreased as the meropenem MIC increased to more than 16 μg/mL. Thus, combinations of a polymyxin plus optimized dosing of a carbapenem appear to be most likely of benefit for treatment of infections caused by CRKP strains with relatively low-level carbapenem resistance (that is, MIC ≤8–16 μg/mL).

### Dual carbapenem therapy

Dual carbapenem therapy refers to the use of ertapenem, which is the carbapenem with the highest affinity for carbapenemase enzymes, in addition to a carbapenem that has lower affinity for the enzyme (for example, meropenem or doripenem) so that ertapenem preferentially consumes the carbapenemase, allowing a larger proportion of the other carbapenem to remain intact and to exert its antimicrobial effect on the infecting organism. *In vitro* or animal models using both immunocompetent and neutropenic mice (or both) have demonstrated enhanced efficacy of dual carbapenem regimens (for example, optimized-dose doripenem plus ertapenem) as compared with a single carbapenem (doripenem) against at least some isolates of KPC- and NDM-producing *K. pneumoniae* ^38, 39^. This strategy appears to be most likely effective for treatment of infections caused by organisms with relatively low carbapenem MICs (for example, ≤4–16 µg/mL) and may be less effective in immunocompromised hosts ^40^. Human clinical data are limited, but at least two small case series of three patients each with high-level CRKP bacteremia or UTI have reported successful treatment using the combination of ertapenem plus either doripenem or meropenem ^41, 42^.

### Optimized dosing of carbapenems

The two previously described treatment strategies both involve the use of optimized dosing of a carbapenem (for example, meropenem or doripenem). This refers to administering the drug in doses and frequencies that are most likely to allow the free drug concentration to exceed the MIC of the infecting organism ( *f*T>MIC) for at least 40% of the dosing interval. Treatment regimens using higher doses and prolonged infusion of carbapenems (for example, meropenem 2 grams intravenously every 8 hours infused over the course of 3 hours) have achieved this *f*T>MIC goal and have been successful in the treatment of infections due to other gram-negative organisms with relatively low-level carbapenem resistance MIC (<16 µg/mL), including *P. aeruginosa*. However, at least one *in vitro* pharmacodynamic model was not reliably able to reach a *f*T>MIC of at least 40% for KPC-producing *K. pneumoniae* isolates with MICs of 8 µg/mL and did not achieve a *f*T>MIC of at least 40% for any isolates with an MIC of 16 µg/mL due to rapid hydrolysis of meropenem ^43^.

### New antimicrobial agents

Ceftazidime-avibactam was approved by the US Food and Drug Administration (FDA) in 2015 for treatment of complicated UTI, including pyelonephritis and, in combination with metronidazole, complicated intra-abdominal infections. Avibactam is a new non-beta-lactam beta-lactamase inhibitor that has activity against class A and C beta-lactamases, including ESBLs, AmpC, and KPC. It is not active against class B metallo-beta-lactamases, such as NDM ^44^. Testing of collections of clinical isolates suggests that ceftazidime-avibactam has activity against most isolates of multidrug-resistant *K. pneumoniae* isolates (FDA breakpoint MIC value ≤8 µg/mL) ^45– 47^. There are currently no data from randomized controlled trials of the use of ceftazidime-avibactam for the treatment of CRKP, but available data from case series suggest that ceftazidime-avibactam may offer a useful therapeutic option for some patients with CRKP infections ^48, 49^. Temkin and colleagues described the use of ceftazidime-avibactam for the treatment of 36 patients with CRE infection (34 of which were caused by CRKP) and two patients with carbapenem-resistant *P. aeruginosa* ^48^. Among those 38 patients, 28 (74%) experienced clinical or microbiological cure or both. Shields and colleagues described their experience using ceftazidime-avibactam to treat 37 patients with CRE infection, 84% of which were *K. pneumoniae* with 90% of those harboring a *bla _KPC_* gene ^49^. Clinical success was achieved in 59% of the patients. Microbiologic failure occurred in 27% of the patients and development of ceftazidime-avibactam resistance (that is, MIC >8 µg/mL) was detected in three 3(30%) of 10 cases within 10–19 days of ceftazidime-avibactam exposure. In subsequent investigations, emergence of mutations in *bla _KPC-3_* was detected among the three isolates that had acquired ceftazidime-avibactam resistance. The resulting variant enzymes were demonstrated to result in resistance to ceftazidime-avibactam; however, these variant enzymes had reduced carbapenamase activity ^50^. Meropenem MICs were reduced by at least 4-fold from baseline with restoration of meropenem susceptibility in two of the three isolates. Other identified mechanisms of resistance to ceftazidime-avibactam among KPC-producing Enterobacteriaceae include production of large amounts of AmpC beta-lactamase ^51^, suggesting that ceftazidime-avibactam may not be active against organisms with this particular combination of beta-lactamases, and mutations in outer-membrane porins (for example, ompK35) ^35, 52^.

Until very recently, the pipeline of new drugs for the treatment of multidrug-resistant gram-negative pathogens had been described as “dry”. However, a number of new drugs with *in vitro* activity against multidrug-resistant strains of *K. pneumoniae* are now in development. These include combinations of existing beta-lactam drugs with new carbapenemase inhibitors and novel drugs within existing drug classes (for example, cephalosporins and aminoglycosides). Table 1 describes drugs that are currently in phase 3 clinical trials or under FDA review or both. Additional investigational drugs are currently being evaluated in phase 1 and phase 2 studies. The development of novel therapeutic agents with activity against multidrug-resistant strains of *K. pneumoniae* and other gram-negative organisms is urgently needed.

**Table 1.** Phase 3 investigational drugs with activity against carbapenem-resistant Enterobacteriaceae.

Investigational drug	Drug class	Comments
Meropenem-vaborbactam	Approved carbapenem (meropenem) plus a beta- lactamase inhibitor (vaborbactam) with activity against serine beta-lactamases, including KPC	Approved by the US Food and Drug Administration in August 2017 for the treatment of complicated urinary tract infections. A trial of meropenem- vaborbactam in patients with serious infections due to confirmed or suspected carbapenem-resistant Enterobacteriaceae is in progress.
Plazomicin	Novel aminoglycoside with greater *in vitro* activity than currently available aminoglycosides against many multidrug-resistant Enterobacteriaceae isolates	
Eravacycline	Semi-synthetic tetracycline with activity against KPC and NDM carbapenemases	
Imipenem/cilastatin- relebactam	Approved carbapenem (imipenem) plus beta-lactamase inhibitor (relebactam) with activity against class A and class C beta-lactamases, including KPC	
Cefiderocol (S-649266)	Siderophore cephalosporin that is more stable against class A (for example, KPC) and class B (for example, NDM, VIM, and IMP) carbapenemases	

IMP, imipenemase metallo-beta-lactamase; KPC, *Klebsiella pneumoniae* carbapenemase; NDM, New Delhi metallo-beta-lactamase; VIM, Verona integron-encoded metallo-beta-lactamase.

## Control and prevention

Given the limited treatment options available for, and the morbidity and mortality associated with, multidrug-resistant *K. pneumoniae* infections, prevention of transmission and subsequent infection with these organisms is critical. In November 2015, the CDC updated their recommendations for CRE prevention and control in acute care hospitals and long-term care facilities that provide skilled nursing or rehabilitation services ^53^. The recommendations reflect the complex, multifactorial epidemiology of CRE transmission in healthcare facilities and include guidance related to surveillance (including active surveillance testing of patients at increased risk of CRE carriage, such as those with recent healthcare exposure outside the US or in regions of the US with a higher incidence of CRE), basic infection prevention strategies (for example, hand hygiene, contact precautions, and environmental cleaning and disinfection), antimicrobial stewardship, education of healthcare personnel, and communication within and between healthcare facilities. Additional recommendations have been provided to specifically address and mitigate the previously described risk of transmission of pathogens, including *K. pneumoniae*, through the use of duodenoscopes ^10, 54^.

The recommendation to ensure strict adherence to basic infection prevention practices such as environmental cleaning, hand hygiene, and contact precautions is supported by a number of studies conducted in the healthcare environment. For example, rates of environmental contamination in hospital rooms housing patients colonized or infected with *K. pneumoniae* have ranged from 5.4 to 67% ^55– 57^. Investigators have also assessed the frequency at which healthcare workers become contaminated with *Klebsiella* while providing care to patients colonized or infected with these organisms. Overall rates of contamination of gowns or gloves (or both) of 10.4 to 16.9% have been reported ^56^, but further evaluation is needed to identify specific activities as well as patient- and organism-specific characteristics that may be associated with higher rates of contamination.

Most recommended prevention strategies are focused on preventing transmission of CRKP from colonized and infected patients to others. The relatively high rate of subsequent CRE infection among those who are asymptomatic carriers of CRE highlights the current lack of and need for effective interventions to prevent progression to active infection among persons who are carriers. At least one small study has evaluated the effect of a selective digestive decontamination (gentamicin and polymyxin E oral gels and solutions administered four times per day for seven days) on rates of rectal culture positivity among hospital patients with gastrointestinal carriage of CRKP. In this randomized, controlled, double-blind trial, investigators found that significantly more patients in the active treatment arm had negative rectal cultures at 2 weeks (61.1% versus 16.1%, OR 0.13, *P*<0.01) ^58^. The difference between groups was no longer statistically significant at 6 weeks (58.5% versus 33.3%). While such a strategy has the potential to be useful for CRKP-colonized patients during a period of particularly high risk for invasive infection, the results of these data do not seem to support routine use of this strategy given its less-than-reliable efficacy (nearly 40% remained culture-positive at 2 weeks), the potential risk for promoting the development of resistance to polymyxin, which remains an important drug for the treatment of CRKP infections, and the lack of data on the clinical impact of such treatment. Prevention of CRKP infections among carriers of the organism is an area in great need of further research.
